# CT‐based radiomics model to predict spread through air space in resectable lung cancer

**DOI:** 10.1002/cam4.6496

**Published:** 2023-09-07

**Authors:** Jialin Gong, Rui Yin, Leina Sun, Na Gao, Xiaofei Wang, Lianmin Zhang, Zhenfa Zhang

**Affiliations:** ^1^ Department of Lung Cancer, Tianjin Lung Cancer Center, Tianjin Medical University Cancer Institute and Hospital National Clinical Research Center for Cancer, Key Laboratory of Cancer Prevention and Therapy, Tianjin's Clinical Research Center for Cancer Tianjin China; ^2^ School of Biomedical Engineering & Technology Tianjin Medical University Tianjin China; ^3^ Department of Pathology, Tianjin Medical University Cancer Institute and Hospital National Clinical Research Center for Cancer, Key Laboratory of Cancer Prevention and Therapy, Tianjin's Clinical Research Center for Cancer Tianjin China

**Keywords:** contrast‐enhanced CT, lung cancer, operation type, radiomics, spread through air space

## Abstract

**Background:**

Spread through air space (STAS) has been identified as a pathological pattern associated with lung cancer progression. Patients with STAS were related to a worse prognosis compared with patients without STAS. The objective of this study was to establish a radiomics model capable of forecasting STAS before surgery, which can assist surgeons in selecting the most appropriate operation type for patients with STAS.

**Method:**

There were 537 eligible patients retrospectively included in this study. ROI segmentation was performed manually on all CT images to identify the region of interest. From each segmented lesion, a total of 1688 features were extracted. The tumor size, maximum tumor diameters, and tumor type were also recorded. Using Spearman's correlation coefficient to calculate the correlation and redundancy of elements, and redundant features less than 0.80 were removed. In order to reduce the level of overfitting and avoid statistical biases, a dimension reduction process of the dataset was conducted to decrease the number of features. Finally, a radiomics model included 44 features was established to predict STAS. To evaluate the performance of the model, the receiver operating characteristic (ROC) curve was used, and the area under the curve (AUC) was calculated, and the accuracy of the model was verified by 10‐fold cross‐validation.

**Results:**

The incidence of STAS was 38.2% (205/537). The tumor type, maximum tumor diameters, and consolidation tumor ratio were significantly different between STAS group and non‐STAS group. The training group included 430 patients, while the test group was consisted with 107. The training group achieved an AUC of 0.825 (sensitivity, 0.875; specificity, 0.621; and accuracy, 0.749) and the test group had an AUC of 0.802 (sensitivity, 0.797; specificity,0.688; and accuracy, 0.748). The 10‐fold cross‐validation had an AUC of 0.834.

**Conclusion:**

CT‐based radiomic model can predict STAS effectively, which is of great importance to guide the selection of operation types before surgery.

## 1 INTRODUCTION

Spread through air spaces (STAS) was an aggressive pattern of lung cancer, especially non‐small lung cancer (NSCLC), which was defined by the WHO in 2015 as having the air spaces of the surrounding lung parenchyma filled by micropapillary cluster, solid nests, or single cells beyond the edge of the tumor body.^1^ STAS is most commonly found in lung adenocarcinoma (LUAD), and it can also appear in lung squamous cell carcinoma (LUSC), small cell lung cancer (SCLC), and neuroendocrine carcinoma.^2^, ^3^, ^4^ Numerous studies have indicated that the existence of STAS correlates with a poorer prognosis with regard to both overall survival (OS) and recurrence‐free survival (RFS) among patients diagnosed with lung adenocarcinoma.^5^, ^6^, ^7^ Therefore, it is critical to precisely distinguish STAS to choose an appropriate operation for patients with lung cancer.

Due to the prevalent utilization of thin‐section computed tomography (CT) and the increased detection rate of early‐stage NSCLC,^8^ STAS has gradually become known and understood in academia. Several previous studies have reported that STAS usually presents as solid or subsolid nodules in CT images and is correlated with factors such as maximum tumor diameters (Tdmax), solid component diameters, and consolidation tumor ratio (CTR).^9^, ^10^, ^11^ Nevertheless, the interpretation of CT images relies highly on the diagnostic experience of doctors. Radiomics can help analyze medical images and transform them into quantifiable multidimensional data, which presents the possibility of objectively identifying lesions at both the macro and microlevels. As a result, radiomics helps reduce the level of subjectivity in quantifying images and subsequently helps doctors to perform a more comprehensive qualitative analysis of tumor phenotype.^12^ Numerous studies have indicated that radiomics exhibits high levels of stability and reproducibility.^13^, ^14^ This study aimed to create a radiomics model that could forecast the presence of STAS in a retrospective cohort, which can determine the STAS status before surgery and provide guidance on the choice of an appropriate operation type.

## 2 MATERIALS AND METHODS

### 2.1 Patient selection

We retrospectively collected clinicopathological data and contrast‐enhanced thin‐section CT images of 585 consecutive patients undergoing surgery for lung cancer at Tianjin Medical University Cancer Institute and Hospital. The exclusion criteria were as follows: (1) pathology was benign tumor (*n* = 5); (2) preoperative neoadjuvant treatment (*n* = 8); and (3) incomplete data (*n* = 35). Finally, the cohort comprised a total of 537 patients, consisting of 265 males and 272 females, with an average age of 60.7 years. The ethics committee of Tianjin Medical University Cancer Institute and Hospital granted approval for this study (bc2022082).

### 2.2 CT image collection and segmentation

All enrolled patients completed contrast enhanced thin‐section CT scans, and all CT scans were performed by spiral CT scanners (Siemens SOMATOM Definition AS+ and Siemens SOMATOM Drive). The equipment settings for the scan included a detector collimation width of 64 × 0.6 mm, a tube voltage of 120 kVp, and automatic adjustment of the tube current. The images were reconstructed with either a 1.5 mm slice thickness and 1.5 mm gap or a 1.5 mm slice thickness and 1.0 mm gap. The reconstruction matrix was 512 × 512 pixels. The Digital Imaging and Communications in Medicine (DICOM) images were retrieved from the Picture Archiving and Communication System (PACS) and subsequently imported into open‐source 3D‐slicer software (version 4.11) for further analysis.

Two professional doctors (GJL and YR, with 4 years and 10 years of experience in chest radiology, respectively) assessed all the CT images on 3D slicer to determine the tumor type (pure ground‐glass opacities [pGGO], mixed GGO [mGGO], or solid nodule) in consensus and segment of the regions of interest (ROI) on each layer of the CT images manually. Neither doctor knew the pathological type or STAS status. The Tdmax of all nodules and the solid component of mGGO were also measured to calculate the CTR. Interobserver agreement was assessed after segmentation, and intraclass correlation coefficients (ICCs) greater than 0.8 were considered to be available for further analysis.

### 2.3 Radiomics feature extraction

The CT images with ROIs were input into the open‐source software Pyradiomics (https://pyradiomics.readthedocs.io/en/latest/index.html) and the radiomic features such as textural, morphological, intensity, law, and wavelet features were automatically extracted. The gray level cooccurrence matrix (GLCM), neighboring gray‐tone difference matrix (NGTDM), gray level size zone matrix (GLSZM), gray level run length matrix (GLRLM), and gray level dependence matrix (GLDM) were the texture features. In total, 1688 features were extracted from each segmented lesion.

### 2.4 Feature extraction and radiomics model building

**FIGURE 1:**
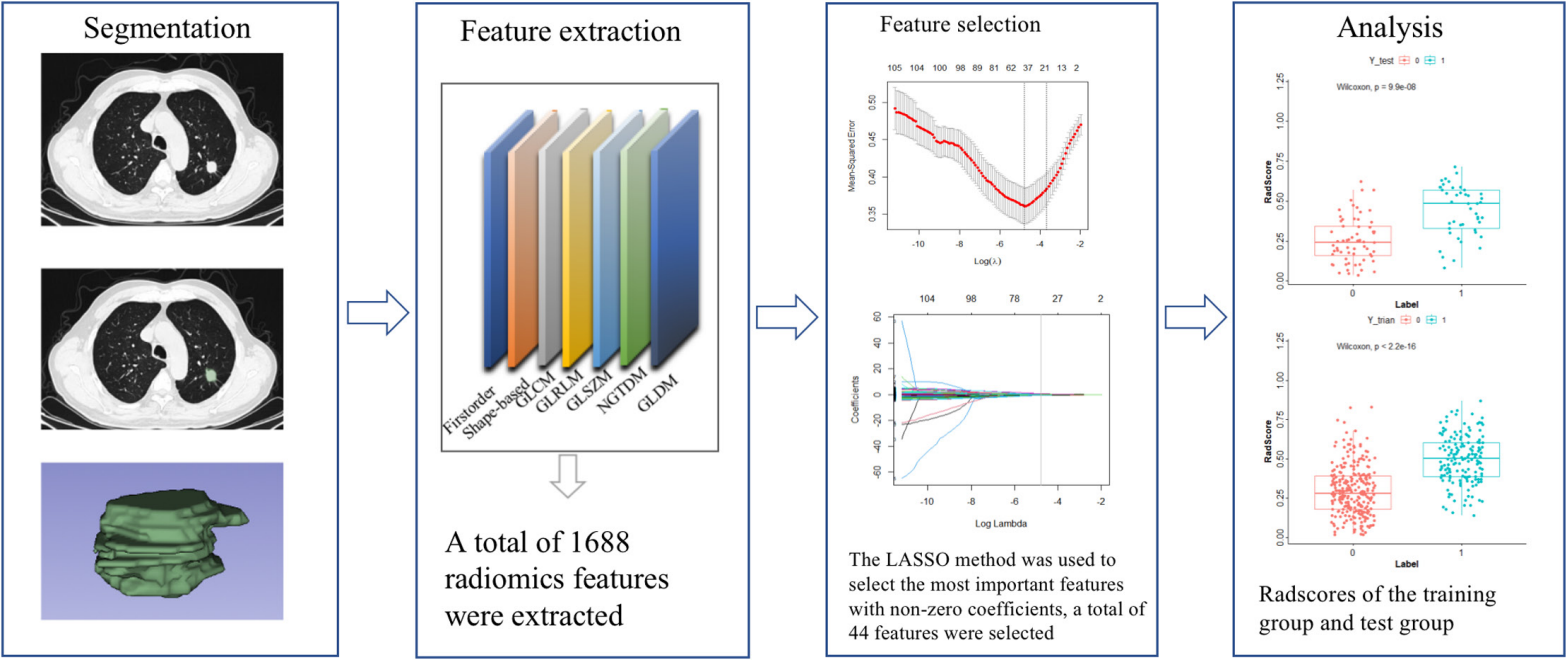
Radiomics workflow.

**FIGURE 2:**
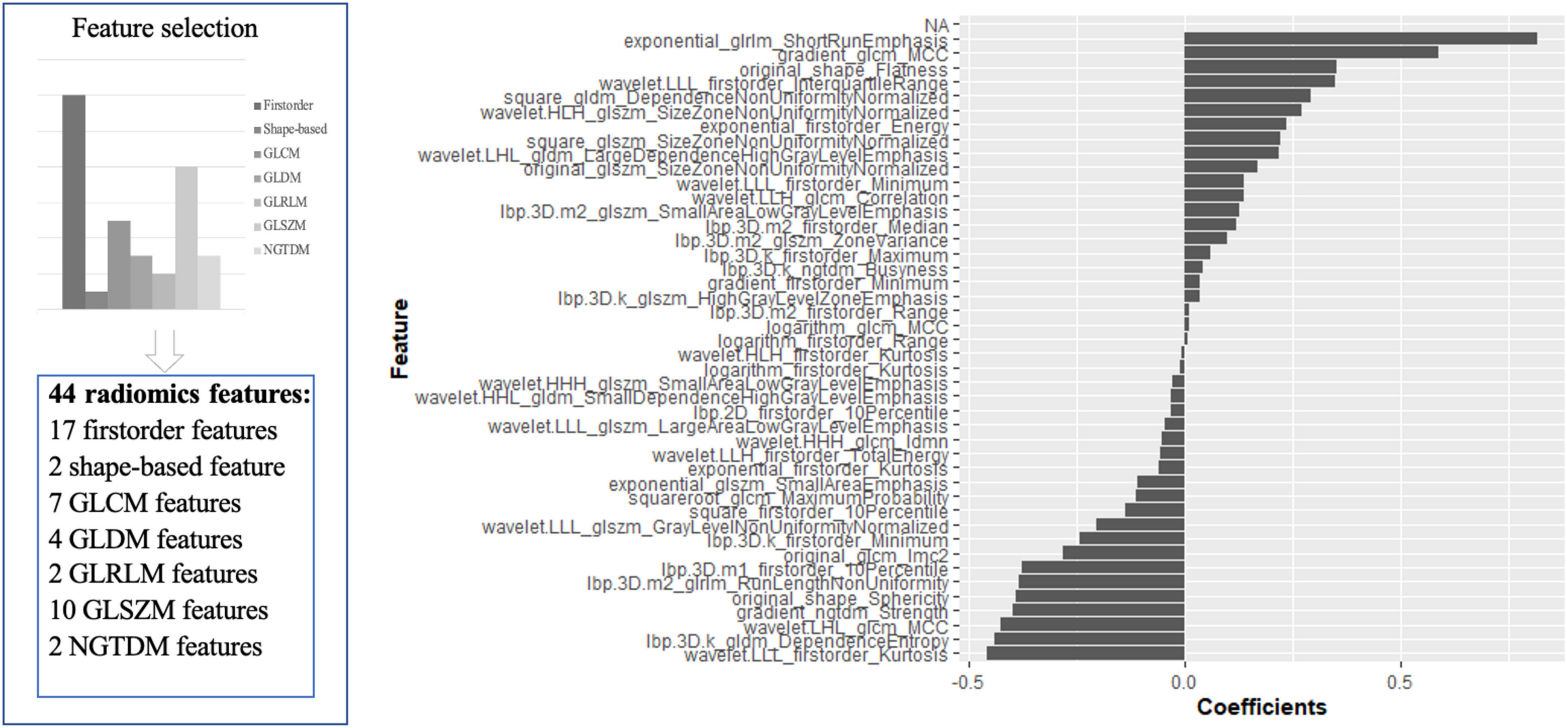
The selected radiomics features.

Spearman's correlation coefficient was used to calculate the association and duplication of elements, and redundant features less than 0.80 were removed. The number of features in the dataset was reduced by applying a dimension reduction process to reduce the level of overfitting and avoid statistical biases. First, the Mann–Whitney U test was used to select features that were highly correlated with STAS. The significance level was set to 0.05 (*p* < 0.05) as the threshold. Second, the interfeature coefficient (R) was calculated for all possible feature pairs to subsequently reduce the dimensionality of the dataset and avoid feature redundancy. The cutoff value for R indicating a strong correlation was set to 0.8. Within a strongly correlated feature pair, the one with a lower *p*‐value would be dropped. Finally, the least absolute shrinkage selection operator method (Lasso) was used to sieve out the most significant features with nonzero coefficients to compute Rad‐scores for all patients. The radiomics workflow is shown in Figure 1, and the selected features are shown in Figure 2.

### 2.5 Histopathological evaluation

Two pathologists (SLN and GN, with more than 5 years and 15 years of experience in thoracic pathology, respectively) who were unaware of the clinical outcomes, conducted evaluations of the hematoxylin–eosin (HE)‐stained tissue sections from all enrolled patients. STAS positivity was defined as tumor cells in airspaces outside the main tumor boundary. STAS positivity was characterized by three distinct morphological patterns: (1) micropapillary structures consisting of papillary structures without central fibrovascular cores; (2) tumor islands or solid nests composed of solid collections of tumor cells filling air spaces; and (3) scattered and discohesive single cells.^5^ Any disagreement was settled through discussion until a consensus was achieved.

**FIGURE 3:**
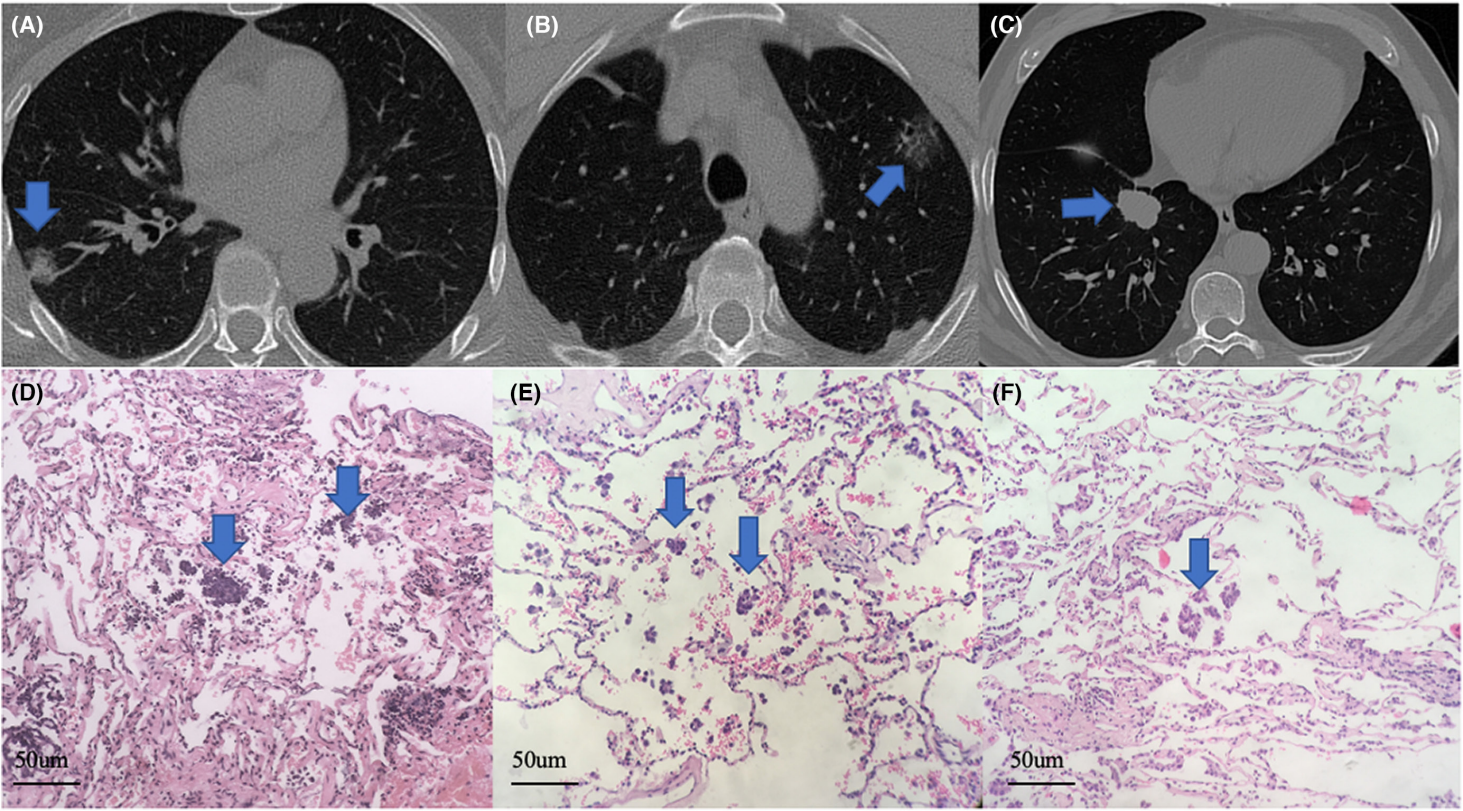
CT images of different tumor types with STAS and hematoxylin–eosin slide of STAS. (A) Mixed density nodule (blue arrow); (B) Pure ground‐glass nodule (blue arrow); (C) Solid nodule (blue arrow); (D–F) Papillary clusters of tumor cells (arrows) in the alveolar space beyond the edge of the main tumor (x20).

We selected typical lung adenocarcinoma with STAS in this study, and the HE slices are shown in Figure 3.

### 2.6 Statistical analysis

The accuracy of the model was assessed through 10‐fold cross‐validation. To evaluate the performance of the model, the area under the curve (AUC) of the receiver operating characteristic (ROC) curve was calculated. The correlation coefficient between the Rad‐score and clinical factors in both the training and test groups was assessed by the Kruskal–Wallis test or Wilcoxon test, as appropriate. Statistical significance was determined by conducting the chi‐square test or Fisher's exact test for categorical variables and the Mann–Whitney test for continuous variables. A *p* value below 0.05 was considered significant. The Rad‐score and features were compared using Student's *t‐*test, and the results are expressed as X ± S. All statistical analyses were conducted using *R* software (version 4.1.0) and IBM SPSS Statistics (version 26.0).

## 3 RESULTS

### 3.1 Clinicopathological features

The clinicopathological features of patients in both the training and test groups are shown in Table 1. There were 205 (38.2%) patients with STAS in this study. The pulmonary nodule density, Tdmax, and CTR were different between the two groups.

**TABLE 1:** Clinicopathological features comparison between training and test group in radiomics.

Variables	Training group, *n* = 430	Test group, *n* = 107	*p*‐value
Age (years)	62 (56, 67)	62 (55, 67)	0.943
Gender, *n* (%)			
Male	210 (48.8)	55 (51.4)	0.667
Female	220 (51.2)	52 (48.6)	
Smoking history, *n* (%)			
Yes	187 (43.5)	57 (53.3)	0.082
No	243 (56.5)	50 (46.7)	
STAS status, *n* (%)			
Yes	160 (37.2)	45 (42.1)	0.356
No	270 (62.8)	62 (57.9)	
Surgical resection, *n* (%)			
Lobectomy	357 (83.0)	88 (82.2)	0.952
Segmentectomy	34 (7.9)	10 (9.3)	
Sleeve resection	4 (0.9)	1 (0.9)	
Wedge resection	35 (8.1)	8 (7.5)	
Tumor location, *n* (%)			
LUL	107 (24.9)	29 (27.1)	0.137
LLL	74 (17.2)	25 (23.4)	
RUL	128 (29.8)	34 (31.8)	
RML	27 (6.3)	7 (6.5)	
RLL	94 (21.9)	12 (11.2)	
Pleura invasion, *n* (%)	72 (16.7)	14 (13.1)	0.382
Cribriform pattern, *n* (%)	27 (6.3)	6 (5.6)	0.830
Vessel invasion, *n* (%)	11 (2.6)	2 (1.9)	
Gene status, *n* (%)			
No mutation	332 (77.2)	78 (72.9)	0.801
EGFR	66 (15.3)	20 (18.7)	
ALK	17 (4.0)	5 (4.7)	
KRAS	12 (2.8)	3 (2.8)	
Others	3 (0.7)	1 (0.9)	
T stage, *n* (%)			
T1a	52 (12.1)	15 (14.0)	0.390
T1b	167 (38.8)	52 (48.6)	
T1c	127 (29.5)	23 (21.5)	
T2a	54 (12.6)	13 (12.1)	
T2b	13 (3.0)	3 (2.8)	
T3	9 (2.1)	1 (0.9)	
T4	8 (1.9)	0	
N stage, *n* (%)			
N0	354 (82.3)	90 (84.1)	0.706
N1	25 (5.8)	4 (3.7)	
N2	51 (11.9)	13 (12.1)	
Pathological type, *n* (%)			
ADC	397 (92.3)	99 (92.5)	0.202
SCC	20 (4.7)	2 (1.9)	
LCLC	6 (1.4)	3 (2.8)	
SCLC	3 (0.7)	0	
Others	4 (0.9)	3 (2.8)	
ADC subtype, *n* (%)			
LPA	135 (31.4)	38 (35.5)	0.546
APA	199 (46.3)	45 (42.1)	
MPA	5 (1.2)	4 (3.7)	
PPA	5 (1.2)	2 (1.9)	
SPA	29 (6.7)	6 (5.6)	
IMA	17 (4.0)	2 (1.9)	
MIA	7 (1.6)	2 (1.9)	
Pulmonary nodule density, *n* (%)			
Solid	187 (43.5)	40 (37.4)	0.029
Pure GGO	71 (16.5)	31 (29.0)	
Mixed	47 (10.9)	8 (7.5)	
Others	125 (29.1)	28 (26.2)	
Tdmax (mm)	22.93 (16.05, 30.87)	19.22 (13.44, 30)	0.020
CTR, *n* (%)			
<0.5	90 (20.9)	33 (30.8)	0.039
>0.5	340 (79.1)	74 (69.2)	

Abbreviations: LUL: left upper lobe; LLL: left lower lobe; RUL: right upper lobe; RML; right middle lobe; RLL: right lower lobe; ADC: adenocarcinoma; SCC: squamous cell carcinoma; LCLC: large cell lung carcinoma; SCLC: small cell lung carcinoma; LPA: lepidic predominant adenocarcinoma; APA: acinar predominant adenocarcinoma; MPA: micropapillary predominant adenocarcinoma; PPA: papillary predominant adenocarcinoma; SPA: solid predominant adenocarcinoma; MIA: microinvasive adenocarcinoma; IMA: invasive mucinous adenocarcinoma.

The comparison of clinical data between patients with and without STAS in the training group is shown in Table 2. Patients were categorized into either the STAS group or non‐STAS group, depending on whether STAS was present or absent. The training group consisted of 430 patients with a median age of 61 years old (IQR: 56–65), and the positive rate of STAS was 37.2% (160/430). No statistically significant differences were observed regarding age, sex, smoking history, gene mutation, or adenocarcinoma subtype between the two groups. There was a significant difference in the operation type (*p* = 0.012) and tumor location (*p* = 0.014) between the STAS and non‐STAS groups. The patients with STAS had a higher likelihood of undergoing lobectomy. The majority of STAS patients were stage T1c (*p* = 0.024) and N0 (*p* < 0.001). In terms of invasive pathological behavior, the patients with STAS in the training group were significantly more likely to have visceral invasion (*p* = 0.023), pleural invasion (*p* < 0.001), and cribriform patterns (*p* < 0.001). Concerning CT features, the STAS group in the training group was more likely to present solid nodules (*p* < 0.001), with a larger nodule diameter (*p* = 0.006) and larger CTR (*p* < 0.001) than the non‐STAS group.

**TABLE 2:** Clinicopathological features in the training group.

Variables	STAS, *n* = 160	Non‐STAS, *n* = 270	*p*‐value
Age (years)	61 (56, 65)	62 (56, 67)	0.272
Gender, *n* (%)			
Male	79 (49.4)	131 (48.5)	0.864
Female	81 (50.6)	139 (51.5)	
Smoking history, *n* (%)			
Yes	69 (43.1)	118 (43.7)	0.907
No	91 (56.9)	152 (56.3)	
Operation type, *n* (%)			
Lobectomy	144 (90.0)	213 (78.9)	0.012
Segmentectomy	6 (3.8)	28 (10.4)	
Sleeve resection	1 (0.6)	3 (1.1)	
Wedge resection	9 (5.6)	26 (9.6)	
Tumor location, *n* (%)			
LUL	35 (21.9)	72 (26.7)	0.014
LLL	36 (22.5)	38 (14.1)	
RUL	40 (25.0)	88 (32.6)	
RML	6 (3.8)	21 (7.8)	
RLL	43 (26.9)	51 (18.9)	
Pleura invasion, *n* (%)	43 (26.9)	29 (10.7)	<0.001
Cribriform pattern, *n* (%)	22 (13.8)	5 (1.9)	<0.001
Visceral invasion, *n* (%)	8 (5.0)	3 (1.1)	0.023
Gene mutation, *n* (%)			
No mutation	132 (82.5)	200 (74.1)	0.345
EGFR	11 (6.9)	55 (20.4)	
ALK	15 (9.4)	2 (0.7)	
Kras	1 (0.6)	11 (4.1)	
Others	1 (0.6)	2 (0.7)	
T stage, *n* (%)			
T1a	9 (5.6)	43 (15.9)	0.024
T1b	54 (33.8)	113 (41.9)	
T1c	59 (36.9)	68 (25.2)	
T2a	22 (13.8)	32 (11.9)	
T2b	8 (5.0)	5 (1.9)	
T3	3 (1.9)	6 (2.2)	
T4	5 (3.1)	3 (1.1)	
N stage, *n* (%)			
N0	112 (70)	242 (89.6)	<0.001
N1	11 (6.9)	14 (5.2)	
N2	37 (23.1)	14 (5.2)	
Pathological type, *n* (%)			
ADC	153 (95.6)	244 (90.4)	0.042
SCC	4 (2.5)	16 (5.9)	
LCLC	3 (1.9)	3 (1.1)	
SCLC	0	3 (1.1)	
Others	0	4 (1.5)	
ADC subtype, *n* (%)			
LPA	36 (23.5)	99 (40.6)	0.399
APA	93 (60.8)	106 (43.4)	
MPA	2 (1.3)	3 (1.2)	
PPA	1 (0.7)	4 (1.6)	
SPA	13 (8.5)	16 (6.6)	
IMA	8 (5.2)	9 (3.7)	
MIA	0	7 (2.9)	
Pulmonary nodule density, *n* (%)			
Solid	94 (58.8)	93 (34.4)	<0.001
Pure GGO	6 (3.8)	65 (24.1)	
Mixed	11 (6.9)	36 (13.3)	
Others	49 (30.6)	76 (28.1)	
Tdmax (mm)	24.54 (18.08, 32.28)	21.53 (15.21, 30.11)	0.006
CTR, *n* (%)			
<0.5	8 (5.0)	82 (30.4)	<0.001
>0.5	152 (95.0)	188 (69.6)	
Rad‐score	0.50 ± 0.15	0.30 ± 0.16	<0.001

Abbreviations: LUL: left upper lobe; LLL: left lower lobe; RUL: right upper lobe; RML; right middle lobe; RLL: right lower lobe; ADC: adenocarcinoma; SCC: squamous cell carcinoma; LCLC: large cell lung carcinoma; SCLC: small cell lung carcinoma; LPA: lepidic predominant adenocarcinoma; APA: acinar predominant adenocarcinoma; MPA: micropapillary predominant adenocarcinoma; PPA: papillary predominant adenocarcinoma; SPA: solid predominant adenocarcinoma; MIA: microinvasive adenocarcinoma; IMA: invasive mucinous adenocarcinoma.

### 3.2 Rad‐score building and selected features to predict STAS

Forty‐four features with nonzero coefficients were chosen to establish the Rad‐score using a LASSO logistic regression model, including 17 first‐order features, seven GLCMs, two GLRLMs, 10 GLSZMs, two NGTDMs, four GLDMs, and two shape features. The details of the features and coefficients are shown in Table S1. The comparison of these features is shown in Table 3.

**TABLE 3:** Radiomics features comparison between STAS and non‐STAS group.

Feature name	STAS *n* = 205	Non‐STAS *n* = 332	*p*
exponential_glrlm_ShortRunEmphasis	0.183 ± 0.554	0.173 ± 0.698	0.064
gradient_glcm_MCC	0.579 ± 0.114	0.535 ± 0.126	<0.001
original_shape_Flatness	0.610 ± 0.128	0.574 ± 0.154	0.004
wavelet.LLL_firstorder_InterquartileRange	1094.157 ± 506.617	893.953 ± 447.582	<0.001
square_gldm_DependenceNonUniformityNormalized	0.136 ± 0.076	0.126 ± 0.087	0.182
wavelet.HLH_glszm_SizeZoneNonUniformityNormalized	0.449 ± 0.031	0.431 ± 0.055	<0.001
exponential_firstorder_Energy	171,670.443 ± 92,233.720	22,999.256 ± 92,233.720	0.349
square_glszm_SizeZoneNonUniformityNormalized	0.404 ± 0.053	0.384 ± 0.066	<0.001
wavelet.LHL_gldm_LargeDependenceHighGrayLevelEmphasis	20,738.233 ± 20,354.318	14,318.529 ± 13,880.744	<0.001
original_glszm_SizeZoneNonUniformityNormalized	0.536 ± 0.042	0.513 ± 0.059	<0.001
wavelet.LLL_firstorder_Minimum	−2922.328 ± 272.112	−2833.476 ± 287.291	<0.001
wavelet.LLH_glcm_Correlation	0.253 ± 0.072	0.235 ± 0.088	0.009
lbp.3D.m2_glszm_SmallAreaLowGrayLevelEmphasis	0.006 ± 0.050	0.000 ± 0.004	0.116
lbp.3D.m2_firstorder_Median	11.030 ± 0.436	10.950 ± 0.528	0.068
lbp.3D.m2_glszm_ZoneVariance	13,931,560.852 ± 90,923,905.709	922,337.204 ± 86,262,758.138	0.338
lbp.3D.k_firstorder_Maximum	4.265 ± 1.207	3.611 ± 1.334	<0.001
lbp.3D.k_ngtdm_Busyness	4036.851 ± 9671.156	2808.112 ± 9702.752	0.154
gradient_firstorder_Minimum	5.278 ± 5.937	8.202 ± 8.338	<0.001
lbp.3D.k_glszm_HighGrayLevelZoneEmphasis	3.912 ± 0.089	3.860 ± 0.151	<0.001
lbp.3D.m2_firstorder_Range	19.858 ± 0.348	19.671 ± 0.432	<0.001
logarithm_glcm_MCC	0.684 ± 0.086	0.674 ± 0.099	0.239
logarithm_firstorder_Range	4767.162 ± 556.488	4556.261 ± 924.418	0.001
wavelet.HLH_firstorder_Kurtosis	5.475 ± 1.693	5.031 ± 1.780	0.005
logarithm_firstorder_Kurtosis	4.405 ± 9.373	18.425 ± 41.093	<0.001
wavelet.HHH_glszm_SmallAreaLowGrayLevelEmphasis	0.014 ± 0.013	0.034 ± 0.074	<0.001
wavelet.HHL_gldm_SmallDependenceHighGrayLevelEmphasis	187.270 ± 119.010	152.399 ± 119.905	0.001
lbp.2D_firstorder_10Percentile	0.251 ± 0.433	0.464 ± 0.530	<0.001
wavelet.LLL_glszm_LargeAreaLowGrayLevelEmphasis	0.031 ± 0.075	0.104 ± 0.841	0.118
wavelet.HHH_glcm_Idmn	0.980 ± 0.010	0.976 ± 0.011	<0.001
wavelet.LLH_firstorder_TotalEnergy	359,002,936.750 ± 430,190,789.280	282,886,520.193 ± 679,869,432.570	0.152
exponential_firstorder_Kurtosis	9.014 ± 27.690	61.895 ± 463.516	0.039
exponential_glszm_SmallAreaEmphasis	0.010 ± 0.075	0.013 ± 0.086	0.710
squareroot_glcm_MaximumProbability	0.005 ± 0.111	0.008 ± 0.013	0.011
square_firstorder_10Percentile	2.053 ± 8.769	10.668 ± 23.842	<0.001
wavelet.LLL_glszm_GrayLevelNonUniformityNormalized	0.011 ± 0.003	0.013 ± 0.005	<0.001
lbp.3D.k_firstorder_Minimum	−1.578 ± 0.045	−1.545 ± 0.061	<0.001
original_glcm_Imc2	0.816 ± 0.081	0.840 ± 0.095	0.003
lbp.3D.m1_firstorder_10Percentile	2.748 ± 0.722	3.060 ± 0.942	<0.001
lbp.3D.m2_glrlm_RunLengthNonUniformity	113.521 ± 75.796	82.569 ± 84.112	<0.001
original_shape_Sphericity	0.662 ± 0.078	0.712 ± 0.082	<0.001
gradient_ngtdm_Strength	1.260 ± 1.623	1.837 ± 1.753	<0.001
wavelet.LHL_glcm_MCC	0.498 ± 0.091	0.523 ± 0.108	0.005
lbp.3D.k_gldm_DependenceEntropy	4.766 ± 0.157	4.681 ± 0.229	<0.001
wavelet.LLL_firstorder_Kurtosis	3.207 ± 1.645	3.781 ± 3.437	0.010

Rad‐score = intercept+βi × Xi. (β: the coefficient of each radiomics feature X: nonzero coefficient radiomics features, i: the sequence number of features; and intercept = −0.853). In this study, the Rad‐score showed a notable increase in the STAS group compared to that in the non‐STAS group (all *p* < 0.001, Table S2).

### 3.3 The correlation between the Rad‐score and clinical factors

**FIGURE 4:**
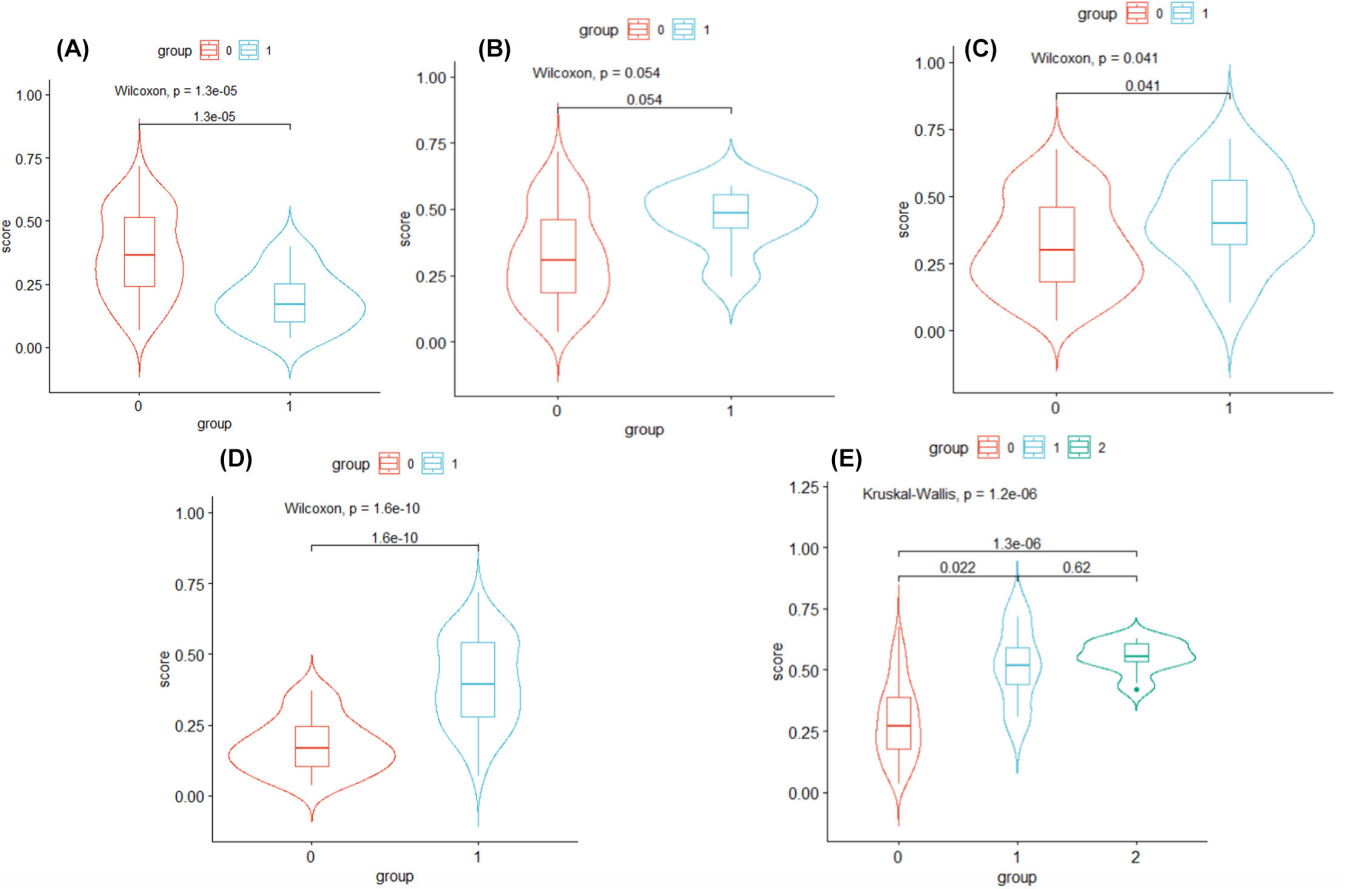
Violin plot. (A) The proportion of lepidic components, 0 = less than 50%, 1 = equal or greater than 50%; (B) Cribriform pattern 0 = no, 1 = yes; (C) Pleura invasion, 0 = no, 1 = yes; (D) CTR, 0 = less than 50%, 1 = more than 50%; (E) N stage, 0 = N0, 1 = N1, and 2 = N2.

We first calculated the correlation between the Rad‐score and each clinical factor in Table 1 in the training group. Subsequently, we selected the significant factors from this analysis for further calculation in the test group. The violin plots are shown in Figure 4.

In the training group, some immunohistochemical markers, such as TTF1, P40, CK56, Naspin A, and Syn, exhibited a significant relationship with the Rad‐score, but this correlation was not significant in the test group. There was a correlation between gene mutations and solid component proportion with Rad‐score in the training group; however, the observed relationship showed no statistically significant results in the testing group (as seen in Figure S1), possibly due to the small sample size. In both the training and test groups, there was a significant correlation between the Rad‐score and CTR, N stage, the proportion of lepidic components, and pleural invasion, with *p* values less than 0.05 for the nonparametric hypothesis tests. The correlation between the Rad‐score and the cribriform pattern had a *p* value of 0.054, indicating a certain degree of correlation.

The radiomics model based on CT images not only had the ability to accurately identify STAS patients but also effectively distinguished patients with CTR values greater than and less than 0.5. Moreover, the model indicated its proficiency in detecting patients with pleural invasion, exhibited a high level of accuracy in identifying patients with advanced N stage, and successfully discerned differences in the proportion of lepidic components.

### 3.4 Radiomics results

**FIGURE 5:**
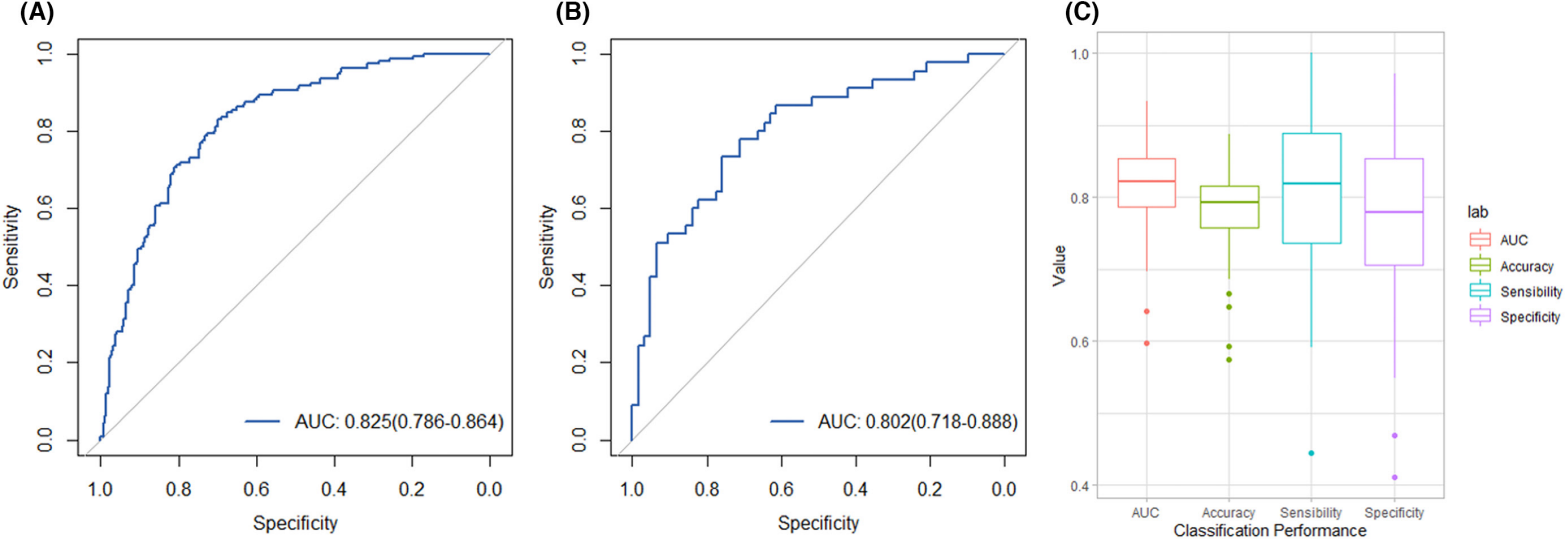
ROC curve for the radiomics model to predict STAS in training group (A) and test group (B). The 10‐fold cross‐validation for the model (C).

The study utilized 44 radiomics features, selected through the feature selection and model building process mentioned earlier, to predict STAS. The coefficients of these features are shown in Figure 2. The model showed satisfactory performance in both the training and test groups, and the AUC was 0.825 in the training group (sensitivity, 0.875; specificity, 0.621; and accuracy, 0.749) and 0.802 in the test group (sensitivity, 0.797; specificity, 0.688; and accuracy, 0.748), as shown in Figure 5. To validate the stability of the model, 10‐fold cross‐validation was conducted, and the AUC was 0.834 (sensitivity, 0.823; specificity, 0.748; and accuracy, 0.778), as shown in Figure 5C.

## 4 DISCUSSION

STAS was first discovered in 2015 and was defined as the presence of micropapillary clusters and/or single cells spreading within air spaces beyond the edge of the main tumor, as well as solid nests.^1^, ^5^ We are confident that STAS is not a random event caused by human factors when obtaining specimens but a real pathological risk factor that reflects the peripheral infiltration of tumor cells.^5^ According to previous studies, the incidence of STAS ranged from 18.2% to 48.1% in lung cancer patients.^5^, ^15^, ^16^, ^17^ The positive rate of STAS in this study was 36.7%. Multiple independent studies have indicated that STAS is predictive of prognosis in all principal histological types of lung cancer.^3^, ^5^, ^7^, ^18^, ^19^, ^20^ STAS is association with lung cancer‐specific death in lung neuroendocrine tumors and squamous cell carcinoma.^3^, ^4^ Dai et al.^7^ studied the impact of STAS and tumor size on survival and found that in lung adenocarcinoma <3 cm, STAS‐positive patients had an unfavorable prognosis. In the 2021 WHO classification of lung tumors, STAS was considered a histologic feature with prognostic significance.^21^ Therefore, finding a method that can predict STAS with good specificity and sensitivity is essential.

Previous studies showed that the radiological factor CTR could preoperatively predict pathological invasive lung cancer with satisfactory specificity among clinical T1N0M0 peripheral patients.^22^ In this study, we found a significant difference in the CTR between the STAS and non‐STAS groups (*p* < 0.001). Furthermore, 95% (152/160) of the STAS patients had a CTR value greater than 0.5, and the results were consistent with the study by Ding and colleagues.^11^ Moreover, a definite correlation between the Rad‐score and CTR was observed, with a Wilcoxon test *p‐*value < 0.01, which suggested a discernible association between STAS and CTR.

In the training group, more than half of the patients had solid nodules in the CT images (*p* < 0.001). Two studies reported a significant correlation between STAS and solid nodules, larger tumor size, micropapillary/solid pattern‐predominant adenocarcinoma, visceral pleural invasion, and lymphovascular invasion.^23^, ^24^ In this study, we also found that pleural invasion, cribriform pattern, and visceral invasion were significantly different between patients with STAS and those without STAS in the training group. The factors mentioned above are invasive characteristics of pulmonary nodules. In this study, pleural invasion and the cribriform pattern were found to have a certain correlation with the Rad‐score, with respective *p* values of 0.041 and 0.054. This finding indicated that our model could effectively identify pleural invasion and, to a certain extent, identify patients with a cribriform pattern. In the field of organ transplantation, when dealing with donor lungs presenting with nodules on CT images, some CT manifestations could aid in estimating the invasive level of the nodules, such as nodule size, maximal CT value, lobulation sign, vessel abnormality,^25^ and STAS. From this perspective, a multiparameter clinical and radiomics model to predict the lung nodule invasive level is highly necessary.

The most common adenocarcinoma subtype in the STAS‐positive group in our study was acinar‐predominant adenocarcinoma, followed by lepidic‐predominant adenocarcinoma and solid‐predominant adenocarcinoma. Similar to previous studies,^17^ the STAS‐positive group had fewer EGFR mutations and more ALK mutations in our study. In summary, the radiological and pathological features of STAS suggest that it may be a potential factor in tumor invasion.

The resection extent of small‐sized pulmonary nodules has always been a hotspot among thoracic surgeons. Lobectomy has been the most commonly used treatment for early‐stage NSCLC, especially after the results of the JCOG0802/WJOG4607L study came out.^26^ The clinical trial JCOG0802 investigated whether segmentectomy was noninferior to lobectomy. The 5‐year overall survival rates for patients who underwent segmentectomy and lobectomy were 94.3% and 91.1%, respectively. The segmentectomy group showed consistently improved overall survival across all predefined subgroups. Based on the results, it can be concluded that segmentectomy is the recommended standard surgical procedure for patients with small‐sized peripheral NSCLC. For STAS‐positive patients, it is crucial to choose an optimal operation type to preserve more lung parenchyma and achieve a better prognosis. Determining which treatment is more suitable for these patients, lobectomy or sublobar, remains a controversial topic. Two groups of researchers conducted separate studies to compare the prognosis of sublobar resection with that of lobectomy for early‐stage IA lung cancer with STAS. Both studies concluded that STAS was an important adverse factor for sublobar resection.^16^, ^27^ Patients with STAS who underwent sublobar resection were found to have a heightened risk of locoregional recurrence, regardless of the margin‐to‐tumor ratio.^27^ Kagimoto et al.^15^ found that in patients with clinical stage IA lung adenocarcinoma and STAS, segmentectomy had a similar prognosis to lobectomy without an increased risk of locoregional recurrence. The optimal operation type is still controversial, so prospective studies investigating which kind of operation type is better for patients with STAS are necessary.

In our study, the radiomics approach showed promising results in predicting the presence of STAS in lung adenocarcinoma as well as in other histological types, such as squamous cell carcinoma, large cell lung cancer, small cell lung cancer, and neuroendocrine cancer. There were 537 patients included in the study, and both the training and testing cohorts were of substantial size. Furthermore, patients with T4 and N2 stages were also included. While several independent studies have established radiomics models to predict STAS, the number of patients enrolled in those studies is relatively small. For instance, Bassi et al.^28^ created a radiomics model to predict STAS using a diverse dataset. Their model demonstrated an accuracy of 0.66 ± 0.02 through internal validation and 0.78 during external validation. Jiang and colleagues^29^ developed a radiomics model based on CT images to predict STAS in lung adenocarcinoma, achieving an AUC of 0.754 despite a relatively low percentage (19.5%) of STAS‐positive tumors in their study. While only 19.5% of tumors were positive for STAS in Jiang's study, the STAS incidence in this study was 36.7%. Additionally, Chen^30^ and Han^17^ also developed radiomics models to predict STAS in stage I lung adenocarcinoma, which indicated good performance.

Nevertheless, the study had a few limitations. First, the data were collected in a retrospective manner from a single institution, which affected the generalizability of the results. To validate the reproducibility and accuracy of our radiomics model, a larger prospective dataset from multiple centers is warranted. Second, without follow‐up data, it is difficult to evaluate the effect of STAS on prognosis and survival after lung cancer surgery. We plan to update the study with follow‐up data in the future.

## 5 CONCLUSION

In conclusion, a significant correlation was found between preoperative CT radiomics features and STAS. We successfully established a CT‐based radiomics model to predict STAS with good performance. This model can serve as a basis for the preoperative diagnosis of STAS and aid in selecting the appropriate operation type in patients with resectable lung cancer.

## AUTHOR CONTRIBUTIONS

**Jialin Gong:** Conceptualization (supporting); data curation (lead); formal analysis (lead); funding acquisition (supporting); investigation (lead); methodology (lead); project administration (supporting); resources (supporting); software (supporting); supervision (supporting); validation (lead); visualization (lead); writing – original draft (lead); writing – review and editing (equal). **Rui Yin:** Conceptualization (supporting); data curation (lead); formal analysis (lead); funding acquisition (supporting); investigation (lead); methodology (lead); project administration (supporting); resources (supporting); software (lead); supervision (supporting); validation (lead); visualization (supporting); writing – original draft (lead); writing – review and editing (equal). **Leina Sun:** Conceptualization (supporting); data curation (supporting); formal analysis (supporting); funding acquisition (supporting); investigation (lead); methodology (supporting); project administration (supporting); resources (lead); software (supporting); supervision (supporting); validation (supporting); visualization (supporting); writing – original draft (supporting); writing – review and editing (equal). **Na Gao:** Conceptualization (supporting); data curation (supporting); formal analysis (supporting); funding acquisition (supporting); investigation (lead); methodology (supporting); project administration (supporting); resources (supporting); software (supporting); supervision (supporting); validation (supporting); visualization (supporting); writing – original draft (supporting); writing – review and editing (equal). **Xiaofei Wang:** Conceptualization (supporting); data curation (supporting); formal analysis (supporting); funding acquisition (supporting); investigation (lead); methodology (supporting); project administration (supporting); resources (supporting); software (supporting); supervision (supporting); validation (supporting); visualization (supporting); writing – original draft (supporting); writing – review and editing (equal). **Lianmin Zhang:** Conceptualization (lead); data curation (supporting); formal analysis (supporting); funding acquisition (lead); investigation (supporting); methodology (supporting); project administration (lead); resources (lead); software (supporting); supervision (lead); validation (supporting); visualization (supporting); writing – original draft (supporting); writing – review and editing (equal). **Zhenfa Zhang:** Conceptualization (supporting); data curation (supporting); formal analysis (supporting); funding acquisition (lead); investigation (supporting); methodology (supporting); project administration (lead); resources (lead); software (supporting); supervision (lead); validation (supporting); visualization (supporting); writing – original draft (supporting); writing – review and editing (equal).

## CONFLICT OF INTEREST STATEMENT

The authors have declared that there are no competing interests.

## ETHICAL STATEMENT

The work was in accordance with the provisions of the Declaration of Helsinki (as revised in 2013). The ethics committee of Tianjin Medical University Cancer Hospital granted approval for this study (bc2022082).

## Supporting information


Figure S1.
Table S1.
Table S2.

## Data Availability

Research data are not shared.
