# Supplementary material for: CT‐based radiomics model to predict spread through air space in resectable lung cancer
Source: Cancer Med. 2023 Sep 7;12(18):18755–66. doi: 10.1002/cam4.6496 (PMC10557899; doi:10.1002/cam4.6496)

**Table S1 The detail information of radiomics features and coefficients.**

| Radiomics feature name | coefficient |
| --- | --- |
| exponential_glrlm_ShortRunEmphasis | 0.81688844 |
| gradient_glcm_MCC | 0.58717466 |
| original_shape_Flatness | 0.35123804 |
| wavelet.LLL_firstorder_InterquartileRange | 0.34669172 |
| square_gldm_DependenceNonUniformityNormalized | 0.28989231 |
| wavelet.HLH_glszm_SizeZoneNonUniformityNormalized | 0.27073238 |
| exponential_firstorder_Energy | 0.23671231 |
| square_glszm_SizeZoneNonUniformityNormalized | 0.21992511 |
| wavelet.LHL_gldm_LargeDependenceHighGrayLevelEmphasis | 0.21831629 |
| original_glszm_SizeZoneNonUniformityNormalized | 0.16659777 |
| wavelet.LLL_firstorder_Minimum | 0.13754419 |
| wavelet.LLH_glcm_Correlation | 0.13606675 |
| lbp.3D.m2_glszm_SmallAreaLowGrayLevelEmphasis | 0.12404052 |
| lbp.3D.m2_firstorder_Median | 0.11730436 |
| lbp.3D.m2_glszm_ZoneVariance | 0.09605965 |
| lbp.3D.k_firstorder_Maximum | 0.05860069 |
| lbp.3D.k_ngtdm_Busyness | 0.04144325 |
| gradient_firstorder_Minimum | 0.03571308 |
| lbp.3D.k_glszm_HighGrayLevelZoneEmphasis | 0.03247809 |
| lbp.3D.m2_firstorder_Range | 0.00895408 |
| logarithm_glcm_MCC | 0.00881433 |
| logarithm_firstorder_Range | 0.00518133 |
| wavelet.HLH_firstorder_Kurtosis | -0.0071599 |
| logarithm_firstorder_Kurtosis | -0.0132891 |
| wavelet.HHH_glszm_SmallAreaLowGrayLevelEmphasis | -0.0282285 |
| wavelet.HHL_gldm_SmallDependenceHighGrayLevelEmphasis | -0.0325293 |
| lbp.2D_firstorder_10Percentile | -0.0335202 |
| wavelet.LLL_glszm_LargeAreaLowGrayLevelEmphasis | -0.045625 |
| wavelet.HHH_glcm_Idmn | -0.0555224 |
| wavelet.LLH_firstorder_TotalEnergy | -0.0578502 |
| exponential_firstorder_Kurtosis | -0.060938 |
| exponential_glszm_SmallAreaEmphasis | -0.1100445 |
| squareroot_glcm_MaximumProbability | -0.1142422 |
| square_firstorder_10Percentile | -0.1400827 |
| wavelet.LLL_glszm_GrayLevelNonUniformityNormalized | -0.2058245 |
| lbp.3D.k_firstorder_Minimum | -0.2431097 |
| original_glcm_Imc2 | -0.2846272 |
| lbp.3D.m1_firstorder_10Percentile | -0.3796322 |
| lbp.3D.m2_glrlm_RunLengthNonUniformity | -0.3866593 |
| original_shape_Sphericity | -0.391249 |
| gradient_ngtdm_Strength | -0.3985896 |
| wavelet.LHL_glcm_MCC | -0.4273435 |
| lbp.3D.k_gldm_DependenceEntropy | -0.4409505 |
| wavelet.LLL_firstorder_Kurtosis | -0.45896 |

**Table S2 Rad-score comparison between the training group and test group**

| Group | STAS | Non-STAS | P value |
| --- | --- | --- | --- |
| Training | 0.50±0.15 | 0.30±0.16 | <0.001 |
| Test | 0.44±0.16 | 0.26±0.14 | <0.001 |

Figure S1 Violin plot A) TTF1; B) P40; C) CK56; D) Napsin A ;E) Syn;0=positive; 1=negative; 2= undetected; F) The proportion of solid components, 0=less than 10%,1= equal or greater than 10%; G) gene mutation, 0=no mutation, 1= EGFR mutation, 2= ALK fusion.


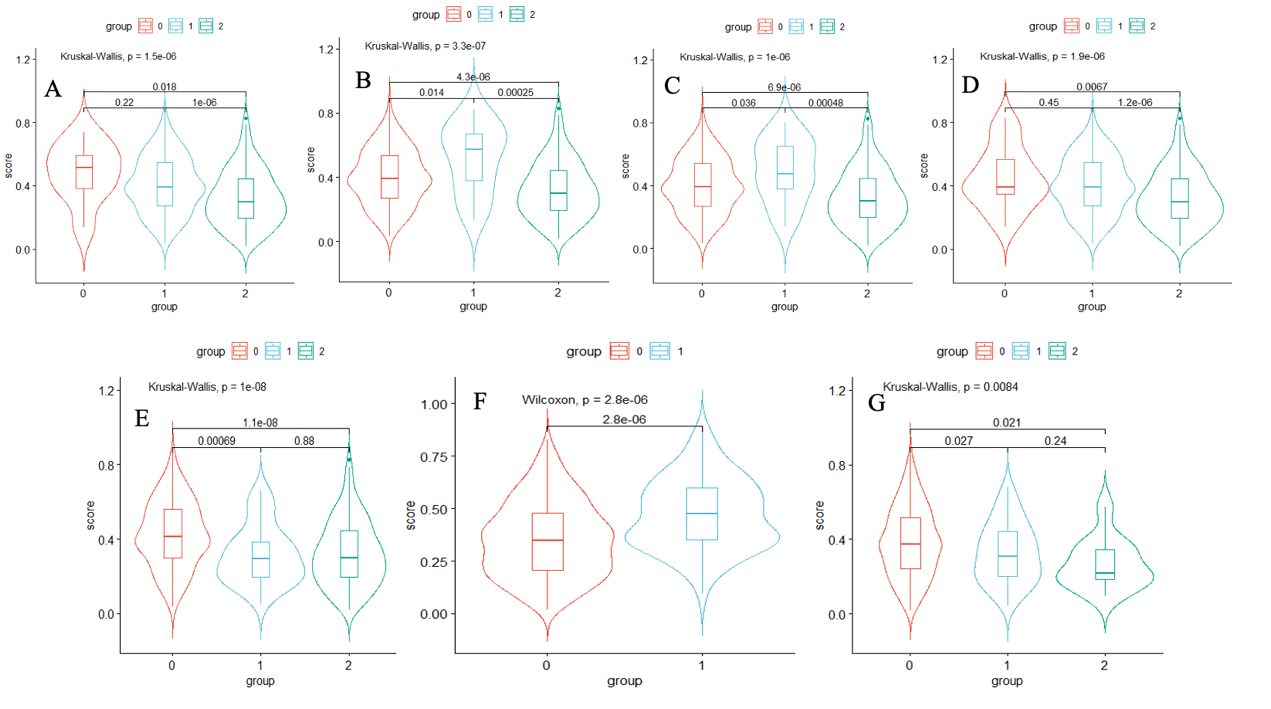

Supplement: Supplementary file 1 — Figure S1. Table S1. Table S2. [file CAM4-12-18755-s001.docx]
